# Psychological Adjustment Profiles of LGBTQ+ Young Adults Residing with Their Parents during the COVID-19 Pandemic: An International Study

**DOI:** 10.3390/ijerph20043188

**Published:** 2023-02-11

**Authors:** Inês Vázquez, Jorge Gato, Susana Coimbra, Fiona Tasker, Jaime Barrientos, Marina Miscioscia, Elder Cerqueira-Santos, Anna Malmquist, Daniel Seabra, Daniela Leal, Marie Houghton, Mikael Poli, Alessio Gubello, Mozer de Miranda Ramos, Mónica Guzmán-González, Alfonzo Urzúa, Francisco Ulloa, Matilda Wurm

**Affiliations:** 1 Faculty of Psychology and Education Sciences, University of Porto, 4200-135 Porto, Portugal; 2 Centre for Psychology, University of Porto, 4200-135 Porto, Portugal; 3 Department of Psychological Sciences, Birkbeck, University of London, London WC1E 7HX, UK; 4 Faculty of Psychology, University Alberto Hurtado, Santiago 6500620, Chile; 5 Department of Women’s and Children’s Health, University of Padua, 35122 Padua, Italy; 6 Department of Developmental Psychology and Socialization, University of Padua, 35122 Padua, Italy; 7 Department of Psychology, Federal University of Sergipe, Aracaju 49100-000, Brazil; 8 Division of Psychology, Linköping University, 581 83 Linköping, Sweden; 9 Center for Research in Neuropsychology and Cognitive and Behavioural Intervention (CINEICC), University of Coimbra, 3000-115 Coimbra, Portugal; 10 Department of Developmental and Family Psychology, Université Libre de Bruxelles, 1050 Brussels, Belgium; 11 School of Psychology, Universidad Católica del Norte, Antofagasta 0610, Chile; 12 MUMS—Movimiento por la Diversidad Sexual, Santiago 578, Chile; 13 School of Behavioral, Social and Legal Sciences, Örebro University, 702 81 Örebro, Sweden

**Keywords:** resilience, LGBTQ+, COVID-19, adjustment profiles, well-being, person-centered approach, social support

## Abstract

The COVID-19 pandemic has been associated with poor mental health symptoms, particularly among vulnerable populations such as LGBTQ+ individuals. In the present study, we aimed to (i) identify different psychological adjustment profiles among LGBTQ+ young adults during the COVID-19 pandemic and compare LGBTQ+ young adults in relation to (ii) sociodemographic characteristics and COVID-19-related experiences and (iii) the internal and external protective resources associated with each adjustment profile. An online questionnaire was administered to 1699 LGBTQ+ young adults from six countries (Brazil, Chile, Italy, Portugal, Sweden, and the UK). A cluster analysis was conducted, and four profiles of psychological adjustment were identified: unchallenged, resilient, distressed, and at-risk. The at-risk cluster scored lowest in social support (particularly from family). The profiles of participants who experienced the highest levels of pandemic adversity (at-risk and resilient) comprised mostly South American participants, those under lockdown at the time of survey completion, those who self-identified as transgender and non-binary, and those with a plurisexual sexual orientation. Interventions should consider strategies to help young adults maintain support systems and reinforce the value of positive family relationships. Specific groups within the LGBTQ+ community that seem to be in a particularly vulnerable situation may need additional tailored support.

## 1. Introduction

The implementation of restrictions to prevent the SARS-CoV-2 virus from spreading (e.g., stay-at-home measures, closure of public services, or teleworking recommendations) had a negative impact on the mental health of most people across and within societies [1,2]. This impact was particularly acute for those who beforehand were in a vulnerable situation [3,4], such as lesbian, gay, bisexual, trans, queer individuals, and persons who self-identified with other sexual and gender minorities (LGBTQ+). In fact, with the beginning of the pandemic, most of the unfavorable living conditions of the LGBTQ+ community were aggravated at a time when the resources of LGBTQ+ rights organizations were depleted. Sources of stress for LGBTQ+ people were reported as (i) loss of income, (ii) diminished access to usual health resources, (iii) potential abuse of governmental powers, (iv) increased stigma and discrimination, and (v) social isolation triggers for domestic violence and loneliness [5]. As a result, studies worldwide reported an increase in psychological distress, depression, anxiety, and substance use among LGBTQ+ individuals [6,7,8,9,10,11,12,13].

Although there is a consensus that the COVID-19 pandemic had a negative impact on the well-being of LGBTQ+ individuals, there is a lack of research focusing on identifying vulnerable groups within this broad community; there is also a lack of protective resources that may have acted as buffers against the impact of the pandemic situation on these individuals’ well-being. Using a resilience framework, our goal in the present study was to identify the variations in the psychological adjustment profiles (low/medium/high) on adaptation outcomes of LGBTQ+ young adults during the COVID-19 pandemic.

### 1.1. Resilience via Internal and External Protective Resources 

Although resilience can be operationalized in various ways, most definitions present a common denominator-positive adjustment despite the presence of risk or adversity thanks to the influence of internal and external protective mechanisms, systems, or resources [14,15,16]. This definition requires the recognition of two distinct dimensions: (i) the presence of situations and/or contexts of risk that threaten psychosocial adjustment; and (ii) the ability to “adjust successfully”. Therefore, positive adjustment is a resilient outcome, and the process of overcoming the risk is resilience [17]. A third component of resilience underlying this construct is the presence of protective mechanisms, i.e., strategies and systems of resources (internal and/or external) that can minimize the effect of risk and increase the probability of psychosocial adjustment [18]. In the current study, we considered LGBTQ+ identity and social support, respectively, as internal and external protective resources, given that both were found to be associated with buffering the effect of various risk factors among the LGBTQ+ population [4,19,20].

#### 1.1.1. LGBTQ+ Identity

The development and integration of an LGBTQ+ identity is often a complex and difficult process. In fact, from a young age, sexual and gender minorities cope with a variety of ongoing stressors related to their sexual and/or gender minority status, including (i) the fear of rejection and victimization, (ii) the need to manage a stigmatized identity, (iii) preoccupation with the degree to which sexual orientation and/or gender identity is accepted by others, (iv) and the challenges of developing a positive identity in a climate of hostility or marginalization [4,21]. Internalized negative attitudes about homosexuality, coupled with worry about the degree to which sexual orientation and/or gender identity will be accepted by others, have been positively correlated with anxiety and depression among LGBTQ+ youths [22,23,24].

Conversely, LGBTQ+ pride—positive attitudes toward sexual orientation and gender identity, greater openness about own sexual and/or gender identity, and greater involvement in the LGBTQ+ community—have been found to be associated with greater psychological adjustment [25,26]. In fact, individuals with a more integrated LGBTQ+ identity have been found to have higher self-esteem and lower levels of stress and anxiety [26,27,28].

#### 1.1.2. Social Support

During the pandemic social support played an important role in preventing mental health problems among the general population [29]. Physical distancing and reduced access to social and community support resources may have limited the availability and reception of social support, which in turn may have exacerbated feelings of social isolation, particularly if social support from family and friends was lacking [30].

In the case of LGBTQ+ individuals, perceptions of social support can provide a sense of validation, social integration, and wholeness that often counterbalance the adverse effects of a negative social climate [21]. During the social restrictions implemented during the COVID-19 pandemic, many LGBTQ+ individuals felt impelled to return to homes unwelcoming of their gender identity and/or sexual orientation, which may have put them at greater risk of exposure to discrimination [5,31]. In fact, parents play a vital role in their LGBTQ+ offspring’s mental health, with the quality of the parent–child relationship and a positive family climate associated with better psychological well-being for LGBTQ+ young adults [19,32]. When families are not supportive, young LGBTQ+ people tend to internalize prejudice and experience difficulties in accepting their own identity, disclosing their identity to others, and engaging with LGBTQ+ community groups [33]. Transgender and non-binary young people appear to be particularly at risk due to a lack of family support [34] and to thrive with LGBTQ+ community support [35]. Not surprisingly, during the COVID-19 pandemic, a less accepting family climate was associated with poor psychological well-being among LGBTQ+ young adults [7,11,32,36,37,38]. 

Sexual and gender minority youths who are rejected by their family may be especially dependent on peer support and be motivated to rely on a wider LGBTQ+ social network [39,40]. By providing LGBTQ+ youths with opportunities to affirm their identities and foster a sense of belonging [41], support and acceptance from friends can be as beneficial as family support [21,42]. During the COVID-19 pandemic, social support from friends was associated with better psychological adjustment when LGBTQ+ youths were compared to peers who received less support from friends [7,11,32,36,39].

### 1.2. The Current Study

Classic studies of resilience have favored the use of person-centered models assuming the individual, not the variables, as the focus of their analyses [43]. Proceeding from a person-centered perspective, such studies have cross-classified people according to their psychological adjustment profiles (e.g., good, medium/mixed, or poor on the criterion for the desired outcome) and multiple risks or challenging conditions (e.g., high, medium/mixed, or low on a criterion of risk or adversity), to explain the differences between these groups [44]. From these classifications, the literature has highlighted four main adaptive or maladaptive profiles: (i) vulnerable/inadequate risk assessment: people with low-risk exposure but with low levels of psychosocial adjustment; (ii) not challenged/normative—individuals inserted in low-risk contexts but with high levels of adjustment; (iii) resilient—people involved in situations of significant adversity and with high levels of adjustment; and (iv) at-risk—individuals who are at high risk and who display evidence of poor psychosocial adjustment [17,44]. 

In contrast, studies focusing on the risk or resilience of LGBTQ+ people have mainly used an analytical approach centered on variables [45]. Moreover, while risk factors have been effectively studied, relatively little is known about the potentially resilient (adaptive) psychological adjustment profiles of sexual and gender minorities. Adaptive (or conversely maladaptive) psychological adjustment profiles may be evident when under general population stress during the COVID-19 pandemic and associated social restrictions. Additionally, in building a person-centered approach to profiling a psychological adjustment, we considered that adjustment is likely to be contingent upon the individual’s local context (e.g., the extent to which social restrictions confine the person to a supportive or unsupportive living environment). Therefore, drawing on a risk-resilience person-centered approach, the present study sought to (i) identify different psychological adjustment profiles among LGBTQ+ young adults during the COVID-19 pandemic, (ii) characterize and examine the differences between profiles according to sociodemographic characteristics and COVID-19 related-experiences, and (iii) compare LGBTQ+ young adults in relation to the internal and external protective resources associated with each adjustment profile. For the operationalization of the objectives, the psychosocial effect of COVID-19 was used as the main adversity indicator. Furthermore, our targeted sampling of LGBTQ+ young adults who resided with their parent/s meant that we focused our adjustment profiling on a subgroup of the LGBTQ+ community identified in previous research as at particular risk of stress. Two mental health outcomes were included as adaptation indicators—anxiety and depression. Finally, both perceived social support (external resource) and LGBT+ identity (internal resource) were considered protective factors in relation to adjustment profiling.

## 2. Methods

### 2.1. Participants

Our convenience sample was composed of LGBTQ+ participants who resided with their parent/s at the time of data collection (N = 1699) from six different countries: Brazil (*n* = 621); Chile (*n* = 482); Italy (*n* = 109); Portugal (*n* = 356); Sweden (*n* = 34); and the United Kingdom (*n* = 97). The participants were aged between 18 and 29 years old (*M* = 22.5, *SD* = 3.27); most resided usually with at least one of their parents (*n* = 1385; 81.5%), and the remainder had returned to reside with their parent/s at some point after the start of the pandemic in their country (*n* = 314; 18.5%). At the time of data collection, most of our samples were totally confined at home (*n* = 1248; 73.5%), some participants were under partial home confinement (*n* = 153; 9%), and the remainder reported not being confined by government stay-at-home orders or social restriction recommendations during the pandemic (*n* = 298; 17.5%). Regarding their gender identity and sexual orientation, most participants were cisgender (*n* =1385; 81.5%) and identified as gay or lesbian (*n* = 802; 47.2%). 

### 2.2. Measures

#### 2.2.1. Sociodemographic Characteristics

The sociodemographic questionnaire included questions about participants’ age, country, sex assigned at birth, gender identity, sexual orientation, residence, relationship status, educational level, professional situation, and religious values. Participants were also asked if they were totally, partially, or not confined in their homes because of government restrictions, if they habitually resided in (or had returned to) their family home, if they were critical workers, and if they were living with a critical worker and/or with a person who was in a designated medically vulnerable group.

#### 2.2.2. Psychosocial Effects of the COVID-19 Pandemic

The Psychosocial Effects of the Lockdown Situation Scale (PELSS) [32] was used to measure the psychological effects of pandemic situations for LGBTQ+ individuals. This instrument has seven items distributed by three subscales, namely Individual Impact (e.g., “To what extent has the COVID-19 pandemic affected your life?”), Social Isolation (e.g., “To what extent has the COVID-19 pandemic made you feel isolated from your heterosexual or cisgender friends?”), and Negative Family Climate (e.g., “To what extent do you feel “suffocated” because you cannot express your LGBTQ+ identity with your family/the people you live with in the current situation of confinement?”). In addition, the items were also summed to give a total score. Participants rated each item using an 11-point Likert-type scale ranging from 0 to 10 (e.g., 0 = Did not affect me at all; 10 = Affected me severely). Items were first devised in Portuguese and then translated into English. Then, each research team translated and back-translated the items into their language. In this study, the total score of the instrument was used (Cronbach’s alpha = 0.69). Items were summed with a higher score indicating more negative psychosocial effects of the pandemic. 

#### 2.2.3. Indicators of Psychological Well-being 

The Depression and Anxiety subscales of the Depression, Anxiety, and Stress Scales 21-Item Version (DASS-21) [46] were used as indicators of psychological well-being. Each country used the adapted version of the DASS-21 [47,48,49,50,51,52,53]. The anxiety subscale measures physical arousal symptoms, panic attacks, and fear (e.g., I felt I was close to panic). The depression subscale includes symptoms usually associated with negative mood (e.g., I felt downhearted and blue). Participants rated items using a 4-point Likert scale (0 = did not apply to me at all to 3 = applied to me very much or most of the time), and items were summed, with the higher scores indicating a greater negative effect. The authors propose a five-dimensional classification between “normal” (0–7 for anxiety and 0–14 for depression) and “very severe” (20+ for anxiety and 34+ for depression). Cronbach’s alphas for the total sample and each country ranged from good to very good values, 0.77 to 0.93 for depression and 0.72 to 0.89 for anxiety.

#### 2.2.4. Internal Protective Resources 

LGBT Identity. Two subscales from the Lesbian, Gay, and Bisexual Identity Scale (LGBIS) [53], adapted to include trans people, were included to tap into LGBT identity. An adapted Portuguese version of LGBIS was available [54], and translations (coupled with back-translation checks) were conducted from the original English version into the other languages as required. Identity Dissatisfaction, composed of six items (e.g., “I wish I were straight and/or cisgender”), assessed the degree to which individuals were dissatisfied with their LGBT identity. Stigma Sensitivity, comprising three items (e.g., “I often wonder whether others judge me for my sexual orientation and/or my gender identity”), assessed the extent to which individuals experienced anxious expectations of rejection based upon their LGBT identity. Items were rated using a seven-point Likert scale (1 = Strongly disagree; 7 = Strongly agree) and summed, with higher scores indicating higher levels of Identity Dissatisfaction and Stigma Sensitivity, respectively. Cronbach’s alphas for the total sample (and each country individually) ranged from good to very good values—0.84 to 0.92 for Identity Dissatisfaction and 0.74 to 0.83 for Stigma Sensitivity.

#### 2.2.5. External Protective Resources

Perceived Social Support. The Multidimensional Scale of Perceived Social Support (MSPSS) [55] was used to assess the subjective perception of social support. Each country used the adapted version of MSPSS [56,57,58,59]. The instrument comprises 12 items (e.g., “My ____ really tries to help me”) with items distributed across three target groups (family, friends, and significant others), rated using a seven-point Likert scale (1 = Strongly disagree; 7 = Strongly agree). Cronbach’s alphas for the total sample and each country yielded a range of very good values—0.89 to 0.96 for Significant Others, 0.91 to 0.94 for Friends, and 0.89 to 0.93 for Family.

### 2.3. Procedure

Data were collected as part of a larger survey study, “Social support networks and psychological health of young LGBTQ+ individuals during the COVID-19 pandemic”. This study, originally devised in Portugal [32], was replicated in Brazil, Chile, Italy, Sweden, and the UK. A core questionnaire was agreed upon, and online survey portals were set up in each country. This study was advertised on LGBTQ+-oriented websites and social media (e.g., Facebook, Instagram) and promoted with the help of local LGBTQ+ community groups. Data were collected from 17 April to 5 August 2020 in six countries. Each country differed in the local severity of the pandemic and in governmental management policy. Over the period of data collection, considering the total number of deaths per 100,000 inhabitants, the UK had the highest mortality rate, followed by Italy and Sweden [2]; Portugal was the least affected country of the six nations at the time. All governments enacted either voluntary stay-at-home recommendations (Portugal, Brazil, and Sweden) and/or stricter lockdown measures (Italy) or a combination of both regimes (Chile and the UK) at the time of this study [36]. While in Europe, most measures were in place from March to May, in some regions of the South American countries, they were still active at the end of the data collection period. Furthermore, in Brazil, there was active government resistance to adopting actions related to COVID-19, with broad presidential support for misinformation [60].

The confidentiality and anonymity of data were guaranteed in each country by not identifying IP addresses. Upon entering the survey portal, all potential participants were informed of the goal of this study and needed to click through electronic consent options to access the survey questions. Contact details for the academics responsible for the research in each country were provided in case participants had concerns or questions. There were no mandatory answers, and an “exit” or “withdraw” button on each page permitted participants to withdraw from the survey at any time. A debriefing information sheet on where to go for further help (e.g., local LGBTQ+ community support services, COVID-19 and health resources, and, ultimately, a licensed psychologist) was automatically displayed for participants when they finished or exited the online survey. Completing the questionnaire took about 15–20 min, and participation was without monetary compensation. This study was approved by the Ethics Committee of the host institution in each country. 

### 2.4. Data Analysis

Regarding the internal consistency of the measures, Cronbach’s alpha values of 0.60 were considered acceptable, but all values lower than 0.70 were cautiously interpreted [61]. Pearson correlations were performed to observe whether the psychosocial effect of COVID-19 was associated with the well-being measures. The magnitude thresholds considered were small (r = 0.10), moderate (r = 0.30), and large (r = 0.50) [62]. 

Then, to estimate the patterns of psychological adjustment during the COVID-19 pandemic, a cluster analysis was conducted, profiling the individuals regarding adaptation (anxiety and depression) and adversity (psychosocial effects of the COVID-19 pandemic). Firstly, a hierarchical cluster analysis (exploratory)—with the method of nearest neighbor and squared with Euclidian distance interval—was utilized. From a range between two and six possible cluster solutions, the chosen solution followed the criteria of the lesser number of groups and association with the greatest increase of explained variances (measured by changes in R^2^). Finally, the k-means clustering method was used to reallocate each observation to the cluster profile with more similarity [63]. 

Analysis of variance made it possible to explore the mean differences between the adversity, protective, and psychological well-being variables among the different psychological adjustment profiles. To explore the possible associations of different adjustment groups with sociodemographic and pandemic experience data, chi-square statistic was used with Monte Carlo simulation correction [64]. To measure the effect size, Cramer’s V (φ_c_) was used [65]. To compare the means of the clusters with protection mechanisms, we used ANOVA. Finally, some cluster groupings were made on a set of sociodemographic variables for parsimony reasons. All analyses were conducted using the 28th version of the IBM SPSS Statistical Package. 

## 3. Results

Pearson correlations indicated that the psychosocial effect of COVID-19 was, as expected, positively associated with anxiety and depression (see Table 1). These results are comparable with those observed in other samples [66] and support the proposition that the COVID-19 pandemic is an impactful risk mechanism for mental health symptoms. Thus, a person-centered analysis to explore patterns of adjustment to COVID-19 is appropriate. 

Based on a previous study [36], which indicated that participants in Brazil and Chile reported significantly more negative psychosocial effects of the COVID-19 pandemic than those in the four European countries, we divided participants into two groups (0 = South American countries; 1 = European countries). Based upon their similar distributions in psychological effects of COVID-19 and well-being variables, binary transgender and non-binary people were grouped as trans. Again for reasons of distribution similarity on the previously mentioned variables, we combined bisexual and pansexual people into a group labeled as plurisexual. 

**Figure 1:**
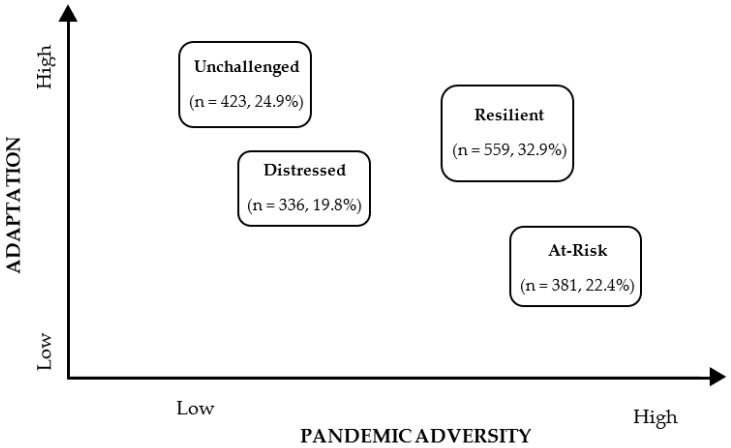
Schematic positioning of the four patterns of adjustment to the psychosocial effect of COVID-19.

The mean psychosocial effect of COVID-19 (*M* = 46.6, *SD* = 12.1) was relatively high within the entire sample. On average, participants manifested a positive internal adjustment with low levels of anxiety (*M* = 7.23, *SD* = 5.12) and depression (*M* = 9.89, *SD* = 5.45). The three variables were standardized for the cluster analysis, with a preliminary analysis revealing that three participants (0.002% of the sample) were outliers (z-score > |3.3|) in at least one of the three measures. Therefore, these three atypical cases were excluded from any subsequent analysis [63]. The hierarchical cluster analysis revealed that the best solution for the data was a four-group clustering solution, with 66.3% of the variance explained (the three-cluster solution explained 53% of the variance, and the five-cluster solution failed to improve explained variance: 67%). After the use of the k-means method for clustering, the four-cluster solution explained 63.8% of the variance. These clusters were statistically different from one another in terms of dimensions of pandemic adversity and adaptation. Results of the ANOVAs and the descriptive statistics of the four clusters, in terms of pandemic adversity and adaptation indicators, are presented in Table 2. For a graphical perspective on the interaction between the levels of adversity and adaptation, see Figure 1. 

### 3.1. Characterization of Psychological Adjustment Profiles

Participants in the most populated cluster presented a high level of the psychosocial effect of COVID-19 and a low level of anxiety and depression; thus, this cluster was named resilient. The unchallenged cluster comprised a high number of participants who endorsed among the lowest levels of psychosocial effect of the COVID-19 pandemic in the sample, alongside some of the lowest levels of negative psychological well-being in the sample. In contrast, the at-risk cluster encompassed the participants with the highest levels of psychosocial effects of the COVID-19 pandemic, as well as high levels of anxiety and depression. Finally, the distressed cluster comprised participants who felt relatively little impact from the pandemic yet who presented moderately high levels of anxiety and depression. 

### 3.2. Psychological Adjustment Profiles and Sociodemographic Characteristics 

A chi-square analysis evaluating sociodemographic characteristics revealed significant differences with small to medium effect sizes between the four psychological adjustment profiles from our cluster analysis with respect to the following variables: continent [χ2 (3) = 144, *p* < 0.001, φ_c_ = 0.291], sex assigned at birth [χ2 (6) = 35.5, *p* < 0.001, φ_c_ = 0.102], gender identity [χ2 (6) = 47.3, *p* < 0.001, φ_c_ = 0.118], sexual orientation [χ2 (12) = 71.9, *p* < 0.001, φ_c_ = 0.119], educational level [χ2 (3) = 22.6, *p* < 0.001, φ_c_ = 0.122], the professional situation [χ2 (6) = 27.7, *p* < 0.001, φ_c_ = 0.090], lockdown situation [χ2 (6) = 82.9, *p* < 0.001, φ_c_ = 0.156], critical worker [χ2 (6) = 24.7, *p* < 0.001, φ_c_ = 0.086], and living with a critical worker and/or a person in a medically vulnerable group [χ2 (6) = 31.9, *p* < 0.001, φ_c_ = 0.097]. For detailed numbers of the participants across the four clusters, see Table 3. Additionally, the analysis of the variance (ANOVA) suggested significant differences between the adaptive profiles regarding age (see Table 4). 

Considering the results of chi-square and the ANOVA, the resilient profile was predominantly composed of South American participants, older individuals, cisgender persons, gay men, and individuals with higher education, employed, in lockdown, and who were living with a critical worker, and/or a person in a medically vulnerable group. The distressed profile, in turn, presented a high number of participants residing in Europe, who were younger, assigned women at birth, transgender and non-binary, heterosexual, plurisexual, and asexual, with 12 years or fewer in education, who were students or unemployed, and not in lockdown. The unchallenged cluster was formed mainly by Europeans, who were older, cisgender men, heterosexual and lesbian/gay, had 12 years or fewer in education, were employed, and were critical workers not in lockdown. The at-risk profile was predominantly composed of participants living in South America, younger individuals, women and intersex, transgender and non-binary, plurisexual and asexual, with higher education, who were students or unemployed, in lockdown, and living with critical worker and/or a person in a medically vulnerable group. There were no significant differences in the type of residence [χ2 (3) = 9.4, *p* = 0.054, φ_c_ = 0.078], religious values [χ2 (15) = 14.7, *p* = 0.470, φ_c_ = 0.117], relationship status [χ2 (3) = 2.94, *p* = 0.400, φ_c_ = 0.042] and habitually reside in (or return to) the family´s home [χ2 (3) = 7.16, *p* = 0.067, φ_c_ = 0.065].

### 3.3. Psychological Adjustment Profiles and Protective Systems

To answer the third objective, we investigated whether the psychological adjustment profiles were differently related to the following variables associated with internal and external protective systems—LGBTQ+ Identity Dissatisfaction, Stigma Sensitivity, and perception of social support (friends, family, significant others). Table 4 shows the differences between the four clusters. 

The analysis of the variance (ANOVA) suggested significant differences between the adaptive profiles regarding all variables (*p* < 0.001). The Post Hoc LSD Test revealed that the at-risk cluster of participants perceived friends’ social support as significantly less likely to be supportive in comparison with those in the resilient, distressed, and unchallenged clusters. Concerning family social support, the unchallenged presented the highest scores indicating considerable social support, followed by the distressed, resilient, and at-risk clusters. Finally, regarding significant other’s social support, unchallenged and distressed participants recorded the highest scores, followed by the resilient and at-risk ones. 

Regarding LGBTQ+ identity, the at-risk cluster reported more identity dissatisfaction than did those in the distressed, resilient, and unchallenged clusters. Likewise, the at-risk cluster indicated the highest scores in relation to stigma sensitivity, while participants in the resilient and distressed clusters scored equally, and the unchallenged cluster recorded among the lowest scores in the sample.

## 4. Discussion

Our study aimed to explore and characterize the different psychological adjustment patterns of LGBTQ+ young adults during the COVID-19 pandemic based on the theoretical framework of risk and resilience. The results portrayed four profiles of psychological adjustment to the COVID-19 pandemic—resilient, at-risk, unchallenged, and distressed— filling the four quadrants we had expected, according to the literature. Furthermore, additional analyses revealed that the interaction between the psychosocial effects of COVID-19 and psychological adjustment patterns of LGBTQ+ young adults living at home is neither independent of the participants’ sociodemographic characteristics and COVID-19-related experiences nor from their varied array of internal or external protective systems (identity dissatisfaction, stigma sensitivity, or perceived social support).

### 4.1. Psychological Adjustment Profiles and the COVID-19 Pandemic

Our person-centered approach using cluster analysis revealed four distinctive patterns of psychological adjustment. When considering the psychosocial effects of the COVID-19 pandemic as an indicator of adversity and anxiety and depression as indicators of adaptation, we identified both patterns of adjustment and maladjustment in the first wave of the pandemic. The cluster analysis highlighted two groups of participants who appeared to be either resilient or at risk in the face of the pandemic adversity. In contrast, many participants indicated that their daily lives were little affected by the pandemic. While some participants experienced little personal impact from the pandemic and whose well-being was generally good (fitting the normal group for anxiety and depression), the unchallenged cluster, the others reported low levels of psychological well-being (fitting the moderate group for anxiety and depression) that appeared to be unrelated to their experience of pandemic adversity (the distressed group). These findings are equivalent to the theoretical considerations of the person-centered approach to risk and resilience [17,44]. The identified groups suggest that not all LGBTQ+ young adults adapt in a homogeneous way to deleterious circumstances that affect the general population, the way the COVID-19 pandemic did. The cluster profiles revealed both high or low patterns of psychological adjustment, both by variations in perceived exposure to pandemic adversity and in interaction with sociodemographic characteristics and with internal and protective resources. 

In the presence of high psychosocial adversity from the COVID-19 pandemic, the resilience process was hindered. Similar results have been reported in the face of other adversities [67]. In our findings, when their internal and external protective resource levels were low, and the sociodemographic variables were less likely to favor high well-being, individuals were unable to overcome the consequences of the pandemic, as illustrated by the at-risk group (fitting the moderate group for anxiety and depression). The resilient group manifested a satisfactory adaptation (fitting the normal group for anxiety and depression) that was, nevertheless, significantly lower than that of their unchallenged peers. Nonetheless, participants in this cluster can be seen as resilient since they manifested “the least damaging of all possible symptoms” [68] (p. 613) in the presence of a moderate level of risk. The resilient and unchallenged profiles comprised a higher number of participants than did the at-risk and distressed clusters, as is typical of survey research reports generally; the others have indicated the difficulties of accessing highly vulnerable low-well-being participants via community surveys [69]. Notwithstanding this caveat regarding survey sampling, our findings here reinforce the belief that resilience is the normative and modal response to trauma [70].

### 4.2. Psychological Adjustment Profile Composition: Who Suffered or Surmounted the First Wave of the COVID-19 Pandemic in Terms of Psychological Well-Being?

The results here reveal that the most salient associations—per the observed effect size —are related to the participant’s continent of residence and prevailing pandemic-related social restrictions in operation at the time of survey completion. South American and currently in-lockdown participants were over-represented in both of the high pandemic adversity experience clusters (at-risk and resilient). These results may have two explanations, based on the differences between the two continents regarding—(i) the acceptance of LGBTQ+ individuals and (ii) the local severity of the COVID-19 pandemic at the time of data collection. Regarding the first aspect, 2019 data from the Pew Research Center [71] showed global differences between South America and Europe in terms of acceptance of same-gender sexual relationships. For example, 94% of people surveyed in Sweden said that homosexuality should be accepted, compared to 67% of respondents in Brazil. Regarding pandemic severity, at the time of data collection, infection rates were still growing in South America amidst an insecure political context [60]. In contrast, COVID-19 infection rates were starting to decrease across Europe [36]. In a longitudinal study with college students, Lathabhavan [72] also found a decrease in the indicators of positive adjustment as the number of deaths and infections worsened. It is also important to highlight that the socioeconomic challenges faced by many participants in South America cannot be neglected in the interpretation. Both Brazil and Chile experienced instability and political changes, a lower human development index, and lower levels of schooling in society than was observable in any of the four European countries we surveyed. Thus, the higher levels of general adversity experienced in South America compared to Europe may have compounded the psychosocial effects of pandemic adversity in the current study. 

Some groups of LGBTQ+ young adults were mainly clustered in lower well-being adjustment profiles (i.e., in the at-risk or distressed clusters), namely, those who identified as trans and/or plurisexual. These groups appear to be in a vulnerable situation: those who were more affected by the psychosocial effects of the pandemic and reported poor psychological well-being (participants in the at-risk cluster); or those who perceived little effect of the pandemic on their daily life but who reported poor psychological well-being (the distressed cluster). This result highlights the multidimensional aspect of risk because, for some individuals, poorer psychological well-being cannot be explained by the psychosocial effect of the COVID-19 pandemic alone. Notably, the sexual and gender minority young adults placed mainly in the distressed cluster may possibly have been exposed to other risks not considered in our analysis [17]. The vulnerable position of LGBTQ+ young adults has been identified in previous studies on the COVID-19 pandemic and in relation to minority stress and other adversities. 

Regarding plurisexual individuals, there is evidence that bisexual people are exposed to more minority stressors and, thus, may experience more mental health problems compared with their lesbian and gay peers [6,73]. Plurisexual individuals are a particularly stigmatized group both within society at large and those who experience marginalization within the LGBTQ+ community, reporting less social support than their monosexual peers [74]. A similar pattern can be found for transgender and non-binary individuals [8,75,76], probably due to challenges associated with gender affirmation both in formal and informal social interactions [77]. The COVID-19 pandemic also has had unique impacts on the transgender and non-binary community and seems to have exacerbated ongoing mental health disparities [10]. In this respect, a decrease in access to gender-affirming health care, as well as transgender-specific support services, has been documented during the pandemic period [11]. Furthermore, many transgender and non-binary individuals who were living according to their gender prior to the emergence of COVID-19 have been confronted with pressures to return to living according to their sex assigned at birth upon moving in with their family [9].

Previous studies have found that people assigned female at birth (who identified either as cis-, transgender, or non-binary) reported being under greater psychological pressure during the pandemic compared to their male-assigned peers [64,78,79,80], probably due to specific sources of stress that exacerbated gender inequalities. Prior to the COVID-19 pandemic, almost 75% of unpaid care and domestic work was performed by females, and during the outbreak, childcare pressures on women increased with the temporary closure of schools and childcare facilities, plus the numbers of parents juggling childcare with working online from home increased [81,82,83]. In addition, during the pandemic, women were losing their jobs at higher rates than men and were less likely to have a financial safety net due to greater job insecurity and lower average wage rates [84].

Achieving a higher educational level and being employed (and, therefore, not currently registered as a student) were among the associated characteristics of participants displaying a resilient psychological adjustment profile. In contrast, those currently registered as a student or in full-time education did not significantly feature in the resilient cluster, although they did populate the at-risk cluster. Many students faced additional challenges, such as the sudden transition to distance learning, uncertainty about their future careers, and, indeed, some worried about whether they would be able to continue their enrollment at college [85]. These additional student concerns, plus the fact that this group of respondents was among the youngest in our sample of young adults, could explain their relatively high levels of perceived pandemic adversity. In our study, younger people were more likely to populate both the distressed and at-risk profile groups. Previous research also has shown that younger cohorts of adults have been shown to display lower psychological well-being scores than older cohorts [86]. Emerging adulthood has been noted as an already stressful period, full of substantial conflicts and instability due to changes in education, living arrangements, and relationships, as well as continuing to be a period of biological and developmental changes [87,88]. While this may be a possible explanation for the prevalence of younger individuals in both the distressed and the at-risk psychological adjustment clusters, it needs to be interpreted with caution as the results reveal a small effect size.

Participants who were unemployed when they completed our survey also were more likely than those who were employed to populate the distressed or the at-risk clusters. Other studies have found that financial instability has been associated with an increased risk of developing mental health problems during the COVID-19 pandemic [89,90,91]. Financial security is likely to be less prevalent among young adults who do not have a regular income, which could trigger economic anxiety [92].

Other authors, such as Wang and colleagues [80], have indicated that people living with a person who could be seen as having been more exposed to the SARS-CoV-2 virus or who was deemed medically more vulnerable to a severe infection arising from it would be more worried about the health consequences of the pandemic. In our study, the participants who lived either with a critical worker or with someone who was in a medically designated vulnerable group fell within our clusters associated with the higher levels of perceived pandemic adversity, namely, the at-risk as well as the resilient profile groups.

### 4.3. Internal and External Protective Resources—How Pandemic Adversity Related to Psychological Well-being of LGBTQ+ Young Adults

Based upon theoretical models of risk resilience, we examined the differential effects of perceptions of the availability of internal and external protective resources on ratings of pandemic adversity to examine patterns in the distribution of participants across the four quadrants of psychological adjustment profiles. Specifically, we considered whether the extent of the perceived availability of internal or external resources could be seen to minimize or maximize the impact of stress [44]. In the current study of LGBTQ+ young adults, we found that the differences between clusters displaying relatively higher levels of psychological well-being (labeled resilient and unchallenged) and those populated by participants with lower psychological well-being scores (the distressed and at-risk profile groups) could be explained by the presence of the internal and external protective resources in the management and perception of risk situations. 

Following key premises from minority stress theory [3,4], we predicted that sexual and gender minority adults who regarded their LGBTQ+ identity as a positive self-attribute would have an important internal protective resource available to draw upon when faced with the pandemic and the imposition of restrictions on in-person social contact. In fact, previous research has found that in the face of other adversities, individuals with a more integrated LGBTQ+ identity presented higher indicators of well-being [26,27,28]. More recently, other authors have highlighted that the absence of social contact during the pandemic can lead some LGBTQ+ youths to ruminate more and question their sexual and/or gender identity [31]. Our findings indicated that those in the at-risk cluster who reported suffering pandemic adversity and displayed higher levels of depression and anxiety scored highest on measures of identity dissatisfaction and stigma sensitivity. 

Results from the current study have highlighted the wider importance of external protective resources, namely, social support not only from family but also from friends and/or a significant other. Interestingly, this pattern of findings was particularly evident in relation to support from family members since individuals who were less exposed to pandemic adversity (those in the unchallenged and distressed profile clusters) showed higher levels of perceived relatives’ social support than those recording higher levels of pandemic adversity (participants classified in the resilient and the at-risk clusters). Thus, having social support from the family seemed to have a prominent effect on the perception of adversity and pointed to family support being a particularly important resource for LGBTQ+ young adults during the first wave of the pandemic. With the COVID-19 outbreak, there was a decrease in face-to-face access to friends, and young adults may have become more reliant on family support [93]. Moreover, despite the increasing importance of friends during emergent adulthood, there is some evidence that individuals’ internal working models of attachment to parents remain critical when under stress [94]. Given the restrictions imposed by governments on social contact during the pandemic, LGBTQ+ young adults may have found more support and safety from parents and other family members than from friends [38]. However, LGBTQ+ young adults who had complicated or fragile family relationships may have been placed in a particularly vulnerable situation. As we have seen in current and previous research, family rejection can be particularly devastating for youths and have deleterious consequences in terms of their mental health [31].

Compared to the psychological adjustment cluster differences observed in patterns of social support from family, perceived social support from friends and/or a significant other appeared not to differentiate psychological adjustment profiles to the same extent. The resilient group of young adults whom we identified through our cluster analysis recorded higher levels of family social support than our at-risk participants. However, participants in both the distressed and unchallenged clusters recorded significantly higher rates of perceived social support from family than the participants in either the resilient or the at-risk cluster profiles. Taken together, our results, therefore, indicated that although family support was a key factor in LGBTQ+ young adults’ well-being profiles, it was neither a decisive consideration for the overall psychological adjustment profile of LGBTQ+ young adults during the first wave of the pandemic nor could it always mitigate low levels of psychological well-being. When compared with participants populating the other three psychological adjustment clusters, those in the at-risk psychological adjustment cluster recorded the lowest levels of support from any of the external protective resources investigated (family, friends, or significant other). Thus, the overall picture from our findings seemed to be consistent with the theoretical framework that perceived social support from any network can act as an external protective resource in the face of adversity [14].

### 4.4. Limitations and Future Directions

While our investigation concentrated on systematically considering the relationship between psychological well-being and pandemic stress within a comprehensive risk resilience theoretical framework across a wide range of LGBTQ+ young adults from six nations, this study is not without limitations. First, by choosing only the perceived psychosocial effects of the COVID-19 pandemic as an indicator of adversity, it was not possible to identify whether LGBTQ+ people were exposed to other adversities, which would probably help to explain the findings associated with the distressed cluster. A full understanding of the resilience processes of LGBTQ+ young adults during the COVID-19 pandemic should include cumulative risks, assets, and resources [95]. Second, further comprehensive mental health assessments, including indices of contact with mental health professionals as well as positive well-being indicators [96], would be useful to go beyond our psychological well-being checklists of depression and anxiety symptoms. Third, given the cross-sectional nature of this study, causality cannot be inferred. Furthermore, resilience is a developmental process. Some adversity exposures may have immediate, acute effects on young adults, but these effects may dissipate relatively quickly or may inoculate against future psychological distress. Other exposures may not be as dramatic but may be chronic and linger over time [17]. Therefore, future research should aim to include a longitudinal design to further investigate the risk resilience process of LGBTQ+ young adults facing the COVID-19 pandemic. Finally, regarding the procedure’s limitations, participation in any online survey is reduced by the ease of access to the Internet. This remains a problem in some isolated locations, especially in lockdown conditions.

## 5. Conclusions

When considering the extent to which the pandemic adversity has impacted gender and sexual minority young adults, our findings indicate that pandemic stress was not uniformly experienced among LGBTQ+ communities across four European and two South American countries. Through adopting a person-centered approach to identifying risk resilience, we employed cluster analysis to distinguish four psychological adjustment profiles (unchallenged, resilient, distressed, and at-risk) within our LGBTQ+ sample. Therefore, professionals working with LGBTQ+ young adults should not assume that because an event is normatively considered negative, it is experienced as harmful by all youths. The variability in psychological adjustment profiles experienced by LGBTQ+ young adults during the COVID-19 pandemic suggests that interventions should be tailored to meet the expressed needs of individuals. Several groups within LGBTQ+ communities may be particularly vulnerable to becoming at risk in terms of their psychological adjustment when faced with an experience of adversity: females, gender minority persons, and plurisexual people. In addition, the young adult’s employment of internal protective resources (their positive or negative feelings about their LGBTQ+ identity) may influence their experience of adversity, as may the availability of external protective resources, i.e., their experience of social support from friends, a significant other, and, in particular, their family members.

## Figures and Tables

**Table 1:** Correlations Between Adversity and Adaptation Variables.

	*n*	Min.	Max.	*M*	*SD*	*Sk*	*Ku*	1	2
1. Psychosocial effect of COVID-19	1699	8	70	46.6	12.1	−0.05	−0.08	-	
2. Anxiety	1699	0	21	7.23	5.12	0.57	−0.45	0.281 ***	-
3. Depression	1699	0	21	9.89	5.45	0.22	−0.87	0.359 ***	0.640 ***

Note. *** *p* < 0.001.

**Table 2:** Means and Standard Deviations of Psychosocial effect of COVID-19, Anxiety, and Depression for Each Cluster.

Measure		Unchallenged	At-Risk	Resilient	Distressed	F (3, 1699)
		(*n* = 423, 24.9%)	(*n* = 381, 22.4%)	(*n* = 559, 32.9%)	(*n* = 336, 19.8%)	
Psychosocial effect of C-19	*M* (*SD*)	34.0 (8.96) ^D^	58.0 (6.49) ^A^	53.2 (5.96) ^B^	38.7 (7.41) ^C^	1035 ***
	z-score	−1.02	0.93	0.54	−0.64	η^2^ = 0.647
Anxiety	*M* (*SD*)	2.97 (2.59) ^D^	13.0 (3.88) ^A^	4.75 (2.89) ^C^	10.2 (3.74) ^B^	841 ***
	z-score	−0.80	1.16	−0.45	0.62	η^2^ = 0.598
Depression	*M* (*SD*)	4.50 (2.83) ^D^	16.2 (3.23) ^A^	8.04 (3.46) ^C^	12.6 (3.63) ^B^	980 ***
	z-score	−0.93	1.21	−0.29	0.54	η^2^ = 0.634

Notes. *** *p* < 0.001; Different letters represent statistically significant different values (*p* < 0.001) and are ordered to show the increase/decrease of values.

**Table 3:** Sociodemographic Characteristics Across Each Psychological Adjustment Profile.

Sociodemographic Characteristics	Unchallenged	At-Risk	Resilient	Distressed	Total
	*n* = 423	*n* = 381	*n* = 559	*n* = 336	
Continent					
South America	216 ^0^	300 ^1^	427 ^1^	160 ^0^	1103
Europe	207 ^1^	81 ^0^	132 ^0^	176 ^1^	596
Sex assigned at birth					
Female	192 ^0^	217 ^1^	266 ^0^	189 ^1^	864
Male	226 ^1^	150 ^0^	290 ^1^	141 ^0^	807
Intersex	5 ^0^	14 ^1^	3 ^0^	6	28
Gender identity					
Cisgender	365 ^1^	278 ^0^	485 ^1^	257 ^0^	1385
Trans	49 ^0^	97 ^1^	72 ^0^	70 ^1^	288
Other	5	4	1 ^0^	7 ^1^	17
Sexual orientation					
Heterosexual	31 ^1^	16 ^0^	6 ^0^	35 ^1^	88
Gay/Lesbian	211 ^1^	163 ^0^	295 ^1^	133 ^0^	802
Plurisexual	152 ^0^	153 ^1^	217	142 ^1^	664
Asexual	8	10 ^1^	5 ^0^	11 ^1^	34
Queer, demisexual, or other	3	4 ^1^	3 ^0^	3	11
Educational qualification					
12 years or less	202 ^1^	180 ^0^	235 ^0^	175 ^1^	792
Higher education	165 ^0^	176 ^1^	275 ^1^	102 ^0^	718
Professional situation					
Student or unemployed	253 ^0^	278 ^1^	355 ^0^	244 ^1^	1130
Employed	135 ^1^	90 ^0^	176 ^1^	76 ^0^	477
Other	33 ^1^	12 ^0^	28	15 ^0^	88
Lockdown					
Yes	275 ^0^	299 ^1^	451 ^1^	223 ^0^	1248
No	63 ^1^	17 ^0^	17 ^0^	56 ^1^	153
Partially	85 ^1^	65	91 ^0^	57	298
Critical worker					
Yes	61 ^1^	40	46 ^0^	31	178
No	338 ^0^	327	507 ^1^	291 ^1^	1463
I would prefer not to say	8 ^1^	7 ^1^	0 ^0^	2 ^0^	17
Living with critical worker					
Yes	160 ^0^	208 ^1^	247 ^1^	123 ^0^	738
No	255 ^1^	170 ^0^	301 ^0^	204 ^1^	930
I would prefer not to say	7	3 ^0^	11 ^1^	7 ^1^	28

Notes. ^0, 1^—Significant association (chi-square statistics): ^0^ = lower frequency of cases observed/expected; ^1^ = higher frequency of cases observed/expected.

**Table 4:** Means and standard deviations of age and internal and external protective resources for each cluster.

Measure	Unchallenged	At-Risk	Resilient	Distressed	F (3, 1699)
Age	23.0 (3.68) ^A^	22.0 (3.28) ^B^	22.7 (3.25) ^A^	22.0 (3.03) ^B^	9.03 ***; η^2^ = 0.016
Perceived Social Support—Friends	20.4 (5.68) ^A^	17.7 (6.98) ^B^	19.4 (6.57) ^A^	19.9 (6.28) ^A^	12.4 ***; η^2^ = 0.021
Perceived Social Support—Family	17.6 (5.78) ^A^	10.8 (5.92) ^D^	13.5 (5.93) ^C^	15.9 (6.19) ^B^	95.6 ***; η^2^ = 0.145
Perceived Social Support—SO	20.2 (5.93) ^A^	17.5 (7.32) ^C^	18.5 (6.82) ^B^	20.3 (6.48) ^A^	16.1 ***; η^2^ = 0.028
LGBT Identity Dissatisfaction	12.2 (7.14) ^B^	14.7 (8.64) ^A^	12.7 (7.59) ^B^	13.3 (7.54) ^B^	8.07 ***; η^2^ = 0.014
LGBT Stigma Sensitivity	10.4 (5.29) ^C^	14.9 (5.24) ^A^	13.1 (5.18) ^B^	12.1 (5.36) ^B^	52.1 ***; η^2^ = 0.085

Notes. *** *p* < 0.001; Different letters represent statistically significant different values (*p* < 0.001) and are ordered to show the increase/decrease of values; SO = Significant Others.

## Data Availability

The datasets for this article are not publicly available to protect the participants’ confidentiality. Requests for datasets should be sent to the corresponding author.
